# A Clinical Perspective on the Antimicrobial Resistance Spectrum of Uropathogens in a Romanian Male Population

**DOI:** 10.3390/microorganisms8060848

**Published:** 2020-06-05

**Authors:** Călin Bogdan Chibelean, Răzvan-Cosmin Petca, Cristian Mareș, Răzvan-Ionuț Popescu, Barabás Enikő, Claudia Mehedințu, Aida Petca

**Affiliations:** 1 George Emil Palade University of Medicine, Pharmacy, Science, and Technology of Targu-Mures, 38 Gheorghe Marinescu str., 540139 Targu-Mures, Romania; calin.chibelean@umfst.ro (C.B.C.); eniko.barabas@gmail.com (B.E.); 2 Department of Urology, Mureș County Hospital, 1st Gheorghe Marinescu str., 540136 Targu-Mures, Romania; 3 “Carol Davila” University of Medicine and Pharmacy, 8 Eroii Sanitari blvd., 050474 Bucharest, Romania; claudia.mehedintu@umfcd.ro (C.M.); aida.petca@umfcd.ro (A.P.); 4 Department of Urology, “Prof. Dr. Th. Burghele” Clinical Hospital, 20 Panduri str., 050659 Bucharest, Romania; dr.razvanp@gmail.com; 5 Department of Laboratory Medicine, Mureș County Hospital, 1st Gheorghe Marinescu str., 540136 Targu-Mures, Romania; 6 Department of Obstetrics and Gynecology, Nicolae Malaxa Clinical Hospital, 12 Vergului str., 022441 Bucharest, Romania; 7 Department of Obstetrics and Gynecology, Elias University Emergency Hospital, 17 Marasti blvd., 011461 Bucharest, Romania

**Keywords:** antimicrobial resistance, AMR, urinary tract infections, UTIs, uropathogens, *E. coli*, *Klebsiella*, *Enterococcus*, Romania

## Abstract

Considering urinary tract infections (UTIs), a significant public health problem with negligible recent research, especially on the male eastern European population, we aimed to determine the antimicrobial resistance rates of uropathogens for the most commonly used antibiotics in urological practice in our country. We conducted a multicenter retrospective study in two different teaching hospitals in Romania, analyzing urine samples from 7719 patients to determine the frequency of incriminating pathogens and their resistance to different antibiotics, in a comparative approach. We determined *Escherichia coli* (35.98%) to be the most common pathogen with the highest sensitivity to amikacin (S = 91.72%), meropenem (S = 97.17%) and fosfomycin (S = 86.60%) and important resistance to amoxicillin-clavulanic ac. (R = 28.03%) and levofloxacin (R = 37.69%), followed by *Klebsiella* spp. (22.98%) with the highest sensitivity to amikacin (S = 78.04%) and meropenem (S = 81.35%) and important resistance to amoxicillin-clavulanic ac. (R = 65.58%) and levofloxacin (R = 45.36%); the most frequent Gram-positive pathogen was *Enterococcus* spp. (19.73%) with the highest sensitivity for vancomycin (S = 93.75%) and fosfomycin (S = 87.5%) and considerable resistance to penicillin (R = 33.52%) and levofloxacin (R = 42.04%). The findings are an important tool in managing UTIs and should be acknowledged as reference research not only for clinicians from Romania but for all physicians treating male UTIs.

## 1. Introduction

Urinary tract infections (UTIs) represent a significant source of infections for both genders, while also being an essential and recurrent cause of urologist’s exams [1]. There is an estimate of more than 200 million cases worldwide annually [2]. UTIs are an important cause of hospitalization for all age groups, but especially for older patients. Furthermore, UTIs are also a substantial source of nosocomial infections [3]. The vast majority of studies on urinary infections are developed on female patients due to the higher rate of incidence in comparison to men; thus, guidelines on UTIs consider studies on female patients as reference, although the anatomical genito-urinary differences are considerable. In terms of morbidity, these are the leading causes affecting people of all ages and involving both genders [4]. These infections are characterized by a high multiplication of a variety of pathogens along the urinary tract, resulting in an inflammatory disease which leads to malfunction of the kidneys or other part of the urinary apparatus [5,6].

We know that urinary tract infection in men younger than 60 years without previous catheterization is an uncommon condition, and it should be carefully managed when diagnosed. Real UTIs in the adult male population have an incidence of approximately 5–8 per year per 10,000, usually due to sexually transmitted infections that are related to the urethra and prostate [7]. With age, the incidence of diagnosed infections significantly increases in the elderly male population [8,9]. Previous studies revealed differences of occurrence in 1000 men based on age group population and reported 0.9 up to 2.4 cases before 55 years and about 7.7 cases after 85 years old [10,11]. Comparing the same age group, the incidence of urinary tract infections in older men is almost half of the frequency in the female population before 80 years old, and it comes to equal percentages afterward [10,11]. The etiology of urinary infections and the sensitivity of the antibiotics used to treat them just like any other infection site is demonstrated to rise and fall from time to time and on different regions [12,13].

UTIs affect more women according to their higher predisposition during a lifetime. Still, with age, men develop some structural and functional modifications that affect the normal voiding, which is mostly responsible for the increased risk of infection in the elderly. More complicated infections appear in older men due to prostate enlargement, which causes bladder obstruction and leads to urinary stasis and the appearance of the disease [7]. Recurrent infection with more virulent microorganisms appears in men with urinary tract abnormalities or when an ineffective treatment does not succeed to eradicate the resistant bacteria [9,14]. The most common causes underlying UTIs in men are represented by benign prostatic hyperplasia, urethral stricture, bladder neck obstruction, bladder stone, bladder tumor, bladder diverticula, prostate cancer, foreign bodies, tight phimosis, and detrusor-sphincter dyssynergia.

According to the Infectious Disease Society of America (IDSA), the local antibiotic resistance profile should be known considering that a urine culture is not always recommended by a physician in primary care, thus permitting the best therapeutic choice [15]. Still, the alarming rise in antibiotics resistance rates among various bacterial strains is caused by the inappropriate duration and dosing of the treatment, indiscriminate prescribing, the vast availability of the over the counter antibiotics to everyone, and their use in the animal industry [16]. The number of multidrug-resistant strains has progressively increased, representing real challenges for the clinician to determine the optimal treatment [17]. Several studies show and prove that antibiotic resistance is increasing every year [18,19].

Different antibiotics are empirically used to treat various diseases. Still, the continuous increasing resistance rates due to drug overuse has become a relevant issue in the medical community worldwide. Lots of antibiotic agents classically used showed high levels of resistance to usual uropathogens. Treating UTIs, in which *E. coli* is the responsible bacteria for the infection, with fluoroquinolones, cephalosporins, or trimethoprim-sulfamethoxazole represents the first line of choice. Previous studies in the UK revealed increased levels of resistance to trimethoprim and penicillin, and these are no longer considered effective as an empirical treatment for urinary infections. Nitrofurantoin is well-concentrated and excreted in the urinary tract and showed a low resistance profile. It can be appropriately used as an empirical treatment for cystitis, but it is not effective in other forms of UTIs because of low tissue penetration.

The most worrying situation is the trend of increasing resistance to fluoroquinolone agents. *E. coli* resistance profile for ciprofloxacin raised from 1 to 20% in the last decades [20]. Recent rates of resistance are responsible for delays in the treatment’s success with increasing morbidity and mortality [21,22]. Geographical resistance patterns are imperative to be appreciated for each area to regain control of the infections. The increase in antibiotic resistance in treating UTIs is a severe health problem issue, especially, but not only, in underdeveloped countries where questionable health education represents a threat to antimicrobial resistance through an alarming increase in consuming antibiotics with or without a prescription [23]. Once with the continuous change in the spectrum of bacteria resistance, it is mandatory to conduct multiple studies aiming to determine the geographical patterns in this very problematic public health issue to determine regional antibiotic susceptibility [17,24].

Recently, The European Center for Disease Prevention and Controls placed Romania between the countries with the lowest resistance rates for antibiotic agents in the European Union but still with one of the highest quota (31.2%) for consumption, just after Greece (34.1%) [25,26]. This should be considered as an alarm signal for the medical community to proceed with their studies for more responsible drug administration [26].

Few studies analyzed the sensitivity and resistance patterns of uropathogens in men, and no one evaluated Romanian patients. The overall prevalence in Romania is unknown, and we believe that it is essential that it is unraveled. Our research, a two-centers approach, aims to highlight the resistance rates and sensitivity profile of common antimicrobial agents used to treat UTIs in the male population in Bucharest—“Prof. Dr. Th. Burghele” Clinical Hospital compared to Mures County Hospital, both medical centers from Romania.

## 2. Materials and Methods

### 2.1. Study Design and Sample Population

This two-centers retrospective study was conducted in a tertiary center in Romania, Bucharest, at “Prof. Dr. Th. Burghele” Clinical Hospital (BCH) and Mures County Hospital (MCH)—between 1 September 2018, and 31 January 2019. For each patient, the written informed consent was obtained. The Local Ethical Committee approved the study design of “Prof. Dr. Th. Burghele” Clinical Hospital (no. 2/2019) and Mures County Hospital (no. 6522/2020).

**Figure 1:**
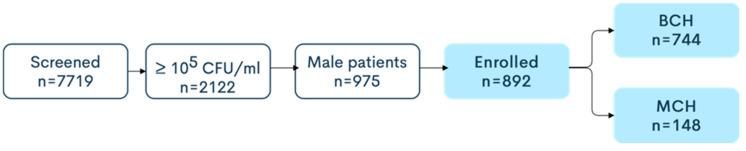
Screening eligibility and enrolment of the study population.

Midstream urine samples from 5293 patients, of which 744 (only males) presented more than 10^5^ CFU/mL, at BCH, were thus selected for evaluation of the type of bacteria and its resistance to current antimicrobial agents used. In MCH, 2426 urine samples were analyzed from 692 patients tested positive (both males and females), of which 148 (only males) met the criteria of selection. We gathered a total of 7719 evaluated urine samples, of which 892 (only males) met the criteria of selection and were further processed—Figure 1.

The selected patients in both clinics were either hospitalized or treated ambulatory. Therefore, a more complex medical history of the non-hospitalized group was unable to be obtained. Moreover, recurrences or first time diagnosed infections cannot be defined. The study also considered information such as the age, sex, or socio-demographic status of each patient.

The inclusion criteria were:

1. Positive uroculture ≥ 10^5^ CFU/mL;
2. Single bacteria strain on uroculture;
3. Age ≥ 18 years;
4. Male patients.

The exclusion criteria were:

1. Uroculture < 10^5^ CFU/mL;
2. Presence of a urological catheter;
3. Multiple bacteria strains on uroculture.

In both hospitals, there was a judicious policy for using antibiotics in the treatment of patients with UTIs, according to Romanian and European guidelines [27,28], which are updated every year. Patients received the minimum therapy course respecting the guideline specification for several pathologies when possible, according to the urine sample results. Different locations and severity of infection imply a particular type of antibiotic management. According to the actual European guidelines, the lower urinary tract infection, such as cystitis, is an unusual diagnosis in men, and it should be considered a complicated infection. A minimum of seven days of course administration with trimethoprim-sulfamethoxazole or fluoroquinolone was considered for higher diffusion in the prostatic tissue. For upper urinary tract infections, such as pyelonephritis, in men, also acknowledged as a complicated disease, the antibiotic strategy should last for 7 to 14 days. The antibiotics preferred were amoxicillin, second- or third-generation cephalosporine combined with an aminoglycoside. Carbapenems were administered whenever the bacterial strain showed antibiotic resistance to the other classes. A lack of previous studies to attest to the fluoroquinolone resistance rate lower than 10% in Romania was an impediment to administer this class.

Examining urine for bacterial load implies a minimum of seven days of time-lapse between testing and the last antibiotic treatment in both centers.

### 2.2. Sample Collection and Processing

The urine collecting was conducted following the International Safety Standards [29]. Urine samples collected in a sterile receptacle were used for culturing the microorganisms on Columbia sheep agar and lactose agar prepared in our laboratory. In additional cases, we used the Chapman medium for *Staphylococcus* spp. Incubation lasted for 24 h at 37 °C for bacteria. Only pure cultures of microorganisms were taken into account; for Gram bacilli, the 10^5^ CFU/mL urine was considered positive. We utilize Vitek 2 automatic microbiology system to determine the density of colonies—CFU/mL [30]. When technical difficulties are encountered due to equipment malfunction, the manual technique is adopted. Consecutively to the Petri dish inoculation of urine specimen, we calculate the bacteriuria according to formula X = N × D × 1/inoculated volume, where X = number of CFU/mL, N = number of colonies on Petri dish, and D = dilution factor (inverse of dilution). The results are filed following the order of magnification of bacteriuria, e.g., <10^3^, 10^3–4^, 10^4–5^, ≥10^5^ CFU/mL [31].

The identification of bacteria was performed based on the morphology of the colonies, biochemical characteristics, such as lactose- positivity or negativity, indole, urease, lysine-decarboxylase, and H_2_S production for enterobacteria. In case of difficulties in differentiating the enterobacteria, we used the Vitek2 automatic system for identification. Gram-staining showed Gram-negative bacilli for the majority of enterobacteria or coccobacilli for *Klebsiella* spp. *Pseudomonas aeruginosa* isolates were diagnosed by morphology, Gram-negative rods, colony-morphologies (pigment production, beta hemolysis, rough colonies with a pleasant smell and metallic shine), oxidase positivity, and non-fermenting characteristics. *Staphylococcus* isolates are Gram-positive cocci that grow well on Chapman medium, where *Staphylococcus aureus* shows yellow, smooth colonies. Pigment production and beta-hemolysis are present on Columbia sheep agar. All staphylococci are catalase positive. Other *Staphylococcus* species were identified by the Vitek 2 automatic system. Enterococci stained Gram positive, and were catalase negative, alpha-hemolysis was present around the smooth, small colonies that proved to grow on agar–bile–esculin medium showing esculin-positive characteristics. Identification on the species level was performed in a few cases with the use of the Vitek 2 automatic system [30].

**Figure 2:**
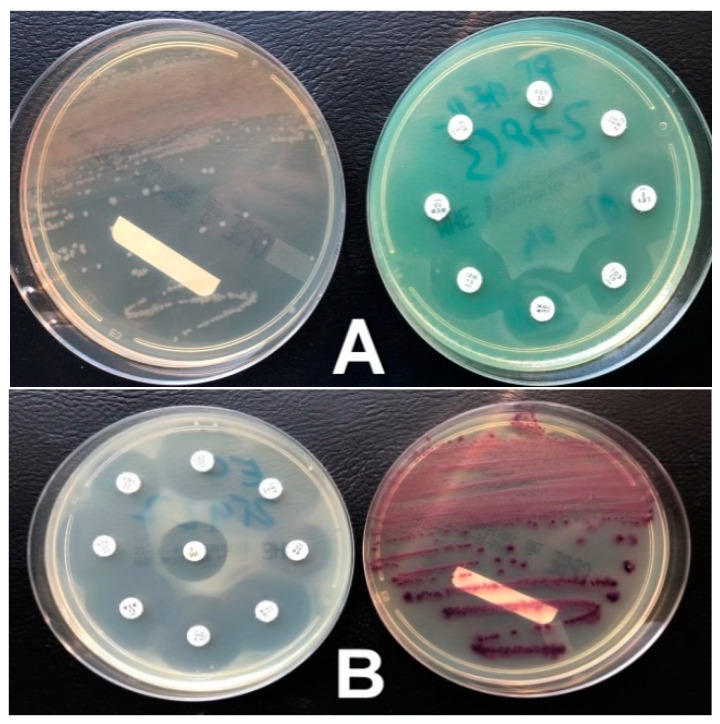
Petri dishes with *E. coli* (**A**) and *Klebsiella* spp. strains (**B**).

We adhered to the Clinical Laboratory Standards Institute (CLSI) guidelines for the disk diffusion technique that we used to determine the antimicrobial susceptibility for each bacterial strain with the adequate antibiotic discs [32]. Pure cultures of the isolates were utilized to prepare the inoculum of 0.5 McFarland in a physiological salt solution. After we swabbed the standard inoculum on the Petri dish, we placed eight antibiotic discs to an equal distance to each other and the same distance to the border of the dish and the ninth antibiotic disc in the center of the plate—Figure 2.

After overnight incubation, we measured the diameters of the inhibition zones according to the disk diffusion technique (Kirby-Bauer) and compared our results with CLSI standards to determine the resistance and the susceptibility of each pathogen agent [32].

### 2.3. Statistical Analysis

The data were analyzed using Microsoft Excel software, and simple descriptive statistics were calculated. Frequency and percentage were determined for certain variables.

## 3. Results

We document a total number of 666 (74.66%) Gram-negative pathogens—of which 542 (72.84%) were in BCH and 124 were (83.78%) in MCH. A total of 226 (25.33%) Gram-positive pathogens were identified—of which 202 (27.15%) were in BCH and 24 (16.21%) were in MCH. Data from both centers and overall statistics are represented in Table 1.

UTIs in men who are not catheterized in any way are not a common discovery among men who are younger than 60 years old, but with an increased incidence among men older than 60 years [33]. In our study, 166 men younger than 60 years old, representing 18.60%, presented a form of urinary infection, while up to 726 men older than 60 years old, representing 81.39%, were diagnosed with UTI. More detailed statistics in patients’ age are presented in Table 2.

In terms of the sensitivity, the most frequent urinary pathogen in BCH, *E. coli*, presented alarmingly high resistance rates for levofloxacin—37.09% resistant strains (R), followed by amoxicillin-clavulanic—R = 28.62%, with lower resistance rates for amikacin R = 4.83%, fosfomycin—R = 0.8%, carbapenems (imipenem and meropenem—R = 0.4%)—Table 3.

**Figure 3:**
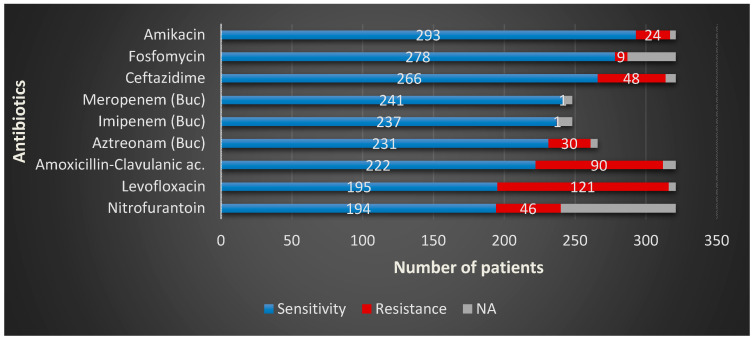
*Escherichia coli* overall sensitivity and resistance profiles in the study group.

In MCH, *E. coli* presented even higher resistance to levofloxacin—R = 39.72%—followed by sumetrolim-sulfamethoxazole—36.98%—ceftazidime—R = 27.39%—amoxicillin-clavulanic ac.—R = 26.02%. The overall two-centers numbers for *Escherichia coli* are represented in Figure 3.

**Figure 4:**
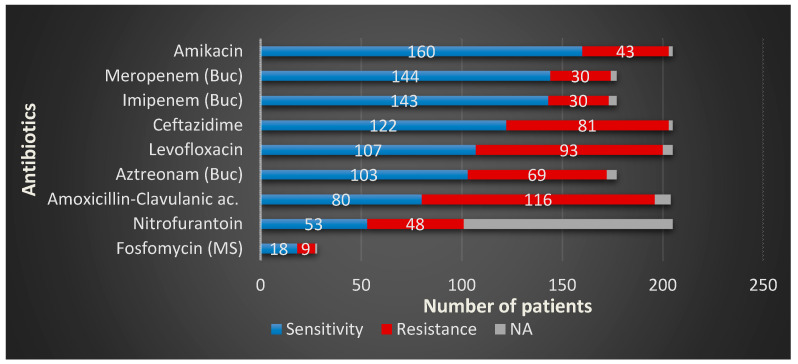
*Klebsiella* spp. overall sensitivity and resistance profiles in the study group.

In BCH, the second most common urinary pathogen, *Klebsiella*, presented alarmingly high resistance rates for most of the antibiotic agents we use. The highest resistance was observed for amoxicillin-clavulanic acid—R = 58.75%—followed by levofloxacin—R = 44.62%—aztreonam—R = 38.98%—ceftazidime—R = 38.41%—nitrofurantoin—R = 22.03%, with the lowest resistance rates registered for carbapenems, imipenem, and meropenem, both R = 1.69%. As in MCH, this particular Gram-negative pathogen presented the highest resistance to levofloxacin—R = 50.0%, followed by ceftazidime—R = 46.42%—amoxicillin-clavulanic ac., both with—R = 42.85%—amikacin—R = 21.14%—and nitrofurantoin—R = 35.71%. The overall numbers for *Klebsiella* are represented in Figure 4.

**Figure 5:**
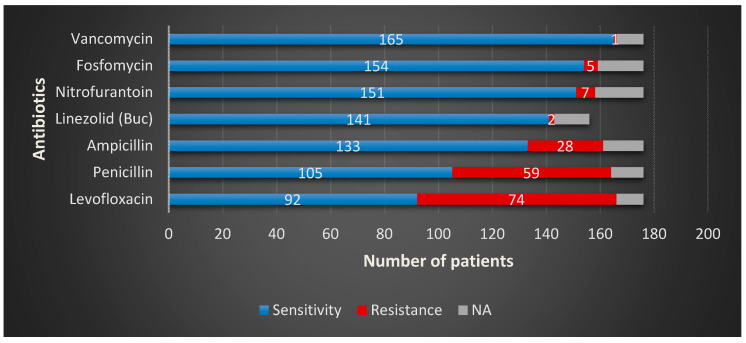
*Enterococcus* spp. overall sensitivity and resistance profiles in the study group.

The resistance rates for *Enterococcus* spp. in BCH to the most common antimicrobial agent used, levofloxacin; its efficiency alarmingly decreased over the past few years. At this moment, almost half of the total amount of strains of *Enteroccocus* proved resistant to it—R = 44.23%—Table 4. It is succeeded by penicillin, with more than one out of three strains resistant to this particular drug—R = 36.53%. It has relatively low resistance to nitrofurantoin—R = 4.48%—Fosfomycin—R = 2.56%—and linezolid—R = 1.28%. In MCH, the highest rate of resistance to *Enterococcus* spp. was also for levofloxacin—R = 25.0%—followed by amoxicillin-clavulanic ac.—R = 15.0%. The overall numbers for *Enterococcus* spp. are represented in Figure 5.

In terms of sensitivity for *Proteus* spp.—Table 3—the highest overall resistance was discovered for amoxicillin-clavulanic ac.—R = 32.0%—followed by levofloxacin—R = 24.0%—and ceftazidime—R = 14.66%. The resistance to imipenem was tested only in BCH—R = 15.15%, and so was for meropenem—R = 15.15% and aztreonam—R = 7.57%. MCH have also tested the resistance rates for fosfomycin—R = 66.66% and nitrofurantoin—R = 33.33%.

*Pseudomonas aeruginosa* showed the highest resistance rates for levofloxacin—R = 44.61%—followed by amikacin—R = 32.30%—and ceftazidime—R = 26.15%. The resistance rates for both carbapenems (imipenem and meropenem)—R = 25.49%—and aztreonam—23.51%—were determined only in BCH, while amoxicillin-clavulanic ac. and nitrofurantoin, both with R = 28.57%, and Fosfomycin—R = 14.28%—were determined only in MCH.

As Table 4 reveals for *Staphylococcus* spp., the overall resistance to various antibiotics were as follows: the highest rate of resistance was presented by penicillin; two-thirds of the total amount of strains proved resistant to this particular drug—R = 66.0%—followed by levofloxacin, with more than a half of the strains resistant to it—R = 54.0%—the combination of sumetrolim-sulfamethoxazole—R = 36.0%—ceftazidime—R = 18.0% and amikacin—R = 4.0%. Surprisingly, none of the tested strains were resistant to nitrofurantoin—R = 0%—with a sensitivity of 74.0%, considering that 26.0% of the isolates were not tested for this drug. It should also be noted that testing for penicillin and nitrofurantoin was done only in BCH.

## 4. Discussions

Urinary infections affect millions of people annually. Due to an important and uncontrolled prescription of antibiotics, rates of resistance against most of the pathogens involved in UTIs in Romania had significantly increased during the last decade, especially for *Pseudomonas*, *Klebsiella*, and *Staphylococcus*. It is advisable that a more justifiable and prudent approach in both therapeutic and prophylactic prescription for UTIs should be critically weighted considering the potential side effects of the overconsumption, especially the antimicrobial resistance. Furthermore, antibiotic susceptibility testing should be evaluated whenever it is necessary, according to the European Association of Urology guidelines to avoid inadequate treatment, which can only lead to antimicrobial resistance (AMR) [28]. These guidelines on UTIs are detailed for each type and location, and specific recommendations for male patients are described. Even if the guidelines are revised each year, the vast majority of the cited references are ten years old, while approximately one quarter are published before 2000. A sum of studies issued before 1980 is evidence-based medicine for today [28]. Newer surveys are required in the field of prevalence, treatment, and AMR for uropathogens in both males and females. The current study’s results confirm the increasing resistance of uropathogens to frequently prescribed antibiotics and the UTIs prevalence over 50 years in males. Even though the development of specific guidelines on male UTIs are not mandatory, larger-scale surveys on AMR, covering different geographical areas are required.

Lifestyle is considered to have a preeminent impact on human health, and it is strongly correlated with the environment. The external factors, such as the increased temperature, have a harmful repercussion on health status [34]. Multiplication rate is negatively influenced by low temperatures for many organisms, viruses, and bacteria. Schvoerer et al. [35] consider that a temperature increase by only 2°C can create more appropriate conditions for pathogens’ proliferation. The increasing average temperature registered, especially in central and eastern Europe, affects the normal, natural life course and creates proper conditions for viral and bacterial spread [34].

Bucharest is the capital city of Romania. It represents home for more than 2 million citizens, with the largest medical university campus, where BCH is considered a cornerstone teaching hospital in the urology field. The MCH stands in Targu Mures city, a considerably smaller town in the heart of Transylvania, which is home for no more than 150,000 citizens. It serves for Mures county region, which has a population of 450,000 people, thus explaining the differences in the number of selected cases. Reporting our findings to the general population in both centers, we notice a significant difference in UTI prevalence, of about 2.6 in Bucharest and 1.53 in Mures, in 1000 individuals. Bucharest is situated at 85m above sea level. The climate here is mild and generally warm and temperate, with an average temperature of 10.8 °C throughout the year. The Mures County lies on higher grounds, at 314m above sea level. Its citizens enjoy a cold and temperate climate, with an average annual temperature of 9.0 °C. During the year, the median temperatures vary in Bucharest by 24.2 °C, while in Mures they differ only by 22.9 °C. These differences in clime conditions, as well as the agglomerated and more polluted capital city, negatively influence the human response to the increasing number of pathogens and exacerbate their virulence. The climate is temperate and varies between mild and cold in most of the eastern European countries, making this survey’s results valuable for the entire region, at least until further alike reports are published. For now, this study is a pioneer for this geographical area.

We report *E. coli* as the most frequent urinary pathogen. Our findings are comparable to what Koeijers et al. published in their study on male UTIs in the Netherlands, Europe [36]. On the other hand, other studies from South America present different data. Jaime L. Rocha et al. have shown data from a tertiary care center, which evaluated the antibiotic resistance patterns of the UTIs [37]. The most frequent urinary pathogen was also *E. coli*, with 66.1% prevalence. The only difference in incidence between the two studied centers was that, in BCH, *Proteus* spp. (8.87%) was more common than *P. aeruginosa* (6.85%) compared to MCH, where *P. aeruginosa* (9.45%) was more common than *Proteus* spp. (6.08%), but the differences were not significant. Moreover, other studies in Europe presented different incidences of uropathogens, data from Bonadio et al. from Italy [38], and Grude et al. from Norway [39].

The incidence of urinary infections among studied patients according to their age, is similar to what other studies from Leiden, the Netherlands, and Kansas City, USA, recently presented [10,11]. UTIs are a widespread source of bacteremia in men over 60 years [40], but directly related death is not a common finding [41]. Asymptomatic bacteriuria is uncommon among young males, but the incidence increases up to 10% in male patients older than 60 years and up to 40% among long-term care facilities’ patients [8]. Getting older is a definitory element in UTIs’ occurrence in men, as we observed a direct connection with age. In both centers, we noted gradually rising cases of UTIs with every age group (Table 2) in agreement with data in the literature [7]. Comparing our results to the incidence in women, we noticed a lower variability with age and a flatter curve of incidence for females. In the study we conducted last year in BCH [17], studying the multidrug resistance of uropathogens in both males and females, we observed a relatively constant incidence in female UTIs during the lifetime.

Probably the most important finding in both studied centers was the alarmingly high resistance to levofloxacin for *E. coli*. In recent years, the resistance rates for fluoroquinolones have risen exponentially. A recent paper from 2019 for Spain [42], which also aimed to detect uropathogens involved in male UTIs, proved high resistance to *E. coli* to fluoroquinolones, similar to our findings. Even on the African continent, a study from Ethiopia [43] suggests even higher rates of resistance to *E. coli* to levofloxacin—R = 55.6%—a finding that indicates, again, that all over the world the resistance rates are going up, especially for fluoroquinolones, due to overprescription. The resistance rates for amoxicillin-clavulanic ac. are also considerable, over a quarter of all strains are resistant to the combination of the aminopenicillin with the inhibitor of beta-lactamase. These numbers are smaller than the results from the USA [44], where the resistance rates for *E. coli* to aminopenicillins were between 37.9% and 42.8%.

Surprisingly, we detected relatively low resistance rates for *E. coli* to amikacin, nitrofurantoin, and fosfomycin. These last two, “old forgotten drugs,” are a good first-line, empiric treatment of choice, also recommended by the guidelines of the European Association of Urology [28]. Research from early 2019 in Australia [45] scrutinized the effectiveness of these antimicrobial agents on resistant strains, concluding that they are a valuable choice for uncomplicated lower UTIs and that the bacterial resistance to these two agents is uncommon. *E. coli* showed the lowest resistance rate for carbapenems (imipenem and meropenem R = 0.4%). An ample Chinese study [46] published in 2018 admitted a satisfactory sensitivity to carbapenems when dealing with UTIs, but still slightly increased compared to recent years.

*Klebsiella* spp. presented alarmingly high resistance rates for many of the most commonly used antibiotic agents in both clinics. The highest overall resistance was observed for amoxicillin-clavulanic acid, which is similar to other findings from Norway [39]. In Ethiopia [43], the sensitivity of *Klebsiella* strains to the combination of amoxicillin-clavulanic acid is higher compared to European countries, with only 22.2% of the total amount being resistant. The second place in terms of resistance is represented by levofloxacin—almost half of the total strains. These numbers are considerably high compared to other studies also from Europe, the United Kingdom [47], and the USA [44]. Different resistance rates of *Klebsiella* to levofloxacin are reported in other European countries. A report from Portugal [48], May 2019, declares a considerably higher sensitivity to this drug compared to our findings, only around 18% of the total amount of the hospital-acquired UTIs resistant to levofloxacin and no more than 12% of the community-acquired strains.

The rising resistance rates all over Europe determined the European Commission (EC) to implement strict conditions in the use of levofloxacin and other fluoroquinolones in urology [49]; therefore, any prescription of this drug should be seriously analyzed. We found that 39.51% of the total tested strains in both centers were resistant to ceftazidime—a third-generation class of cephalosporins, numbers comparable to other non-European countries. In Pakistan [50], the reported resistance to ceftazidime is 33.6%, and for other cephalosporins as follows: ceftriaxone R= 33.8%, cefotaxime R = 33.4%. In more distant countries, such as Taiwan [51], the resistance rates are even lower, only 17.6% of the total proved resistant to ceftazidime.

*Enterococcus* spp., the most frequent Gram-positive uropathogen, presented important resistance to commonly used antibiotics in both hospitals. Data presented in our results are quite similar to other European countries’ findings, as a study from Germany [52] showed similarities in the resistance rates of *Enterococcus* for ampicillin 15%, which is identical to our results. Another paper from Iran [53] acknowledged higher rates of resistance to *Enterococcus* spp. to all antibiotics tested compared to our study: fluoroquinolones 65.4%, ampicillin 28.2%, and penicillin 68.6%. Decreased efficacy facing *Enterococcus* spp. of nearly all antibacterial agents tested should be a sign for the medical community.

A recent paper from Switzerland [54] from ANRESIS (the Swiss Centre for Antibiotic Resistance) presented data from more than 70% of the annual hospitalizations. It aimed to determine the resistance profiles of the most common uropathogens implicated in UTIs, including data for *Proteus* spp. They compared the sensitivity rates from the year 2009 and 2016. We noticed lower resistance rates compared to our study to all tested antibiotics; the most important differences for quinolones—R = 17.6%—and for the third generation of cephalosporins—R = 0.9%. Another study designed in Rome, Italy [55], described that up to 6.6% of *Proteus* strains presented resistance to two or more antimicrobial agents.

Overall, we observed more similarities with results from other European countries regarding uropathogens’ AMR, here including Spain, Norway, and Germany and, surprisingly, Australia. We noted the most significant differences in the prevalence and AMR of strains comparing with surveys made in countries from other continents, but also the UK, Portugal, Italy, and Switzerland. Regular national and international surveillance for several pathogens in different areas may be essential for monitoring the susceptibility and must identify the germs’ resistance patterns to preserve treatment efficacy accurately.

We conducted last year a retrospective study [17] in BCH, which lasted for three months, September-November 2018, which aimed to determine the sensitivity profile of the most common uropathogens implicated in UTIs among patients, both men, and women. The pathogens’ incidence, were relatively similar, excepting the last two less frequent microbial agents. The present study observed *Pseudomonas* spp. as more numerous than *Staphylococcus* spp., which ranked last in the frequency order. The resistance profile in male UTIs presented higher rates for most of the pathogens, revealing for *E. coli*—levofloxacin 37.69% vs. 32.56%—amoxicillin-clavulanic ac. 28.03% vs. 23.91%— ceftazidime 14.95% vs. 10.17%; for *Klebsiella*—amoxicillin-clavulanic ac. 65.58% vs. 52.06%—levofloxacin 45.36% vs. 33.50%—and ceftazidime 39.51% vs. 33.50% (male vs. female). We consider the differences linked to the more prevalent complicated infections in the male population paralleled to the same pathology in female patients. The absence of a survey to observe the dynamics of female UTIs alone in Romania is an essential limitation of this contrast. We intend to obtain these data from another study.

The retrospective manner of our research narrows the clinical information. The patients were either hospitalized or treated ambulatory; therefore, a more detailed medical history was not available. We cannot precisely tell if these infections were community or hospital-acquired, recurrence, or first time diagnosed, either symptomatic or asymptomatic. Our results were paralleled to reliable data from the literature concerning the influence of climate conditions, without an experimental part on this particular topic. These must be considered the limitations of our study.

We acknowledge that the data presented in our study represent a pioneer in the bacteriological survey of male UTIs in Romania, and more research should concentrate on this particular topic. Extending the research, especially in other regional hospitals, to confront the data obtained for a better understanding of the dynamics of microbial agents involved in male UTIs, will grant the future therapeutic success. This process will accomplish the depiction of the sensitivity profile for the most common antibiotics used against them.

The increased AMR in uropathogens should be a determinant cause for all doctors to rearrange their therapeutic strategies for treatment and prevention to improve patients’ chances in this tough challenge. Antibiotic administration should rely, as much as possible, on urine culture results, and, when necessary, empirical treatment would consider the local studies for bacterial prevalence and resistance profile.

## 5. Conclusions

The incidence of diagnosed UTIs in the male population is strongly correlated with age, and it is a more frequent presence after 50 years.

*E. coli* is the most-likely bacterial prevalence in male adults with UTIs. *E. coli* sensitivity for nitrofurantoin and fosfomycin is proper, as there are still acceptable rates of resistance to amikacin and cephalosporins. For *Klebsiella*, amikacin is still a good therapeutic option, as for fluoroquinolones and amoxicillin-clavulanic acid, alarmingly high resistance rates were detected. *Enterococcus* spp. showed worryingly high resistance to levofloxacin and penicillin.

The AMR of uropathogens implicated in male UTIs demonstrates variable dynamics depending on the bacteria, antibiotics analyzed, and geographical coordinates.

## Figures and Tables

**Table 1:** Isolated uropathogens in the study group.

Isolated Bacteria	BCH		MCH		Total	
	*n*	%	*n*	%	*n*	%
Gram Negative	542	72.84	124	83.78	666	74.66
*E. coli*	248	33.33	73	49.32	321	35.98
*Klebsiella* spp.	177	23.79	28	18.91	205	22.98
*Pseudomonas aeruginosa*	51	6.85	14	9.45	65	7.28
*Proteus* spp.	66	8.87	9	6.08	75	8.40
Gram Positive	202	27.15	24	16.21	226	25.33
*Enterococcus* spp.	156	32.70	20	13.51	177	19.73
*Staphylococcus* spp.	46	6.18	4	2.70	50	5.60

*n*—number, %—percentage.

**Table 2:** Uropathogens in male patients of various age groups in Burghele Clinical Hospital (BCH) and Mures County Hospital (MCH).

Isolated Bacteria	BCH								MCH							
	<31		31–50		51–70		>70		<31		31–50		51–70		>70	
	*n*	%	*n*	%	*n*	%	*n*	%	*n*	%	*n*	%	*n*	%	*n*	%
*E. coli*	1	0.13	18	2.41	123	16.53	106	14.24	6	4.05	8	5.4	29	19.59	30	20.27
*Klebsiella* spp.	0	-	11	1.47	67	9.00	99	13.30	3	2.02	4	2.7	13	8.78	8	5.40
*Pseudomonas aeruginosa*	1	0.13	12	1.61	81	10.88	63	8.46	2	1.35	4	2.7	8	5.4	6	4.05
*Proteus* spp.	1	0.13	2	0.26	20	2.68	43	5.77	2	1.35	1	0.67	2	1.35	4	2.7
*Enterococcus* spp.	0	-	5	0.67	19	2.55	26	3.49	2	2.35	1	0.67	5	3.37	6	4.05
*Staphylococcus* spp.	2	0.26	0	-	23	3.09	20	2.68	0	-	1	0.67	2	2.35	1	0.67
Total	5	0.67	48	6.45	333	44.75	357	47.98	15	10.13	19	12.83	59	39.86	55	37.16

*n*—number, %—percentage.

**Table 3:** Overall antibiotic resistance profile in Gram-negative uropathogens.

Antibiotics	Gram-Negative Organism Isolated											
	*Escherichia coli*			*Klebsiella* spp.			*Pseudomonas aeruginosa*			*Proteus* spp.		
	R·n/%	S·n/%	NA	R·n/%	S·n/%	NA	R·n/%	S·n/%	NA	R·n/%	S·n/%	NA
Amikacin	24/7.47	293/91.27	-	43/20.97	160/78.04	-	21/32.30	40/61.53	-	4/5.33	70/93.33	
Amoxicillin-Clavulanic ac.	90/28.03	222/69.15	-	116/65.58	80/39.02	-	4/28.57	5/35.71	51-BCH	24/32.0	45/60.0	
Aztreonam	30/12.0	213/85.88	73- MCH	69/38.98	103/58.19	28-MCH	12/23.52	37/72.54	14-MCH	5/7.57	59/89.39	9-MCH
Ceftazidime	48/14.95	266/82.86	-	81/39.51	122/59.51	-	17/26.15	47/72.30	-	11/14.66	62/82.66	
Fosfomycin	9/2.80	278/86.60	-	9/32.14	18/64.28	177-BCH	2/14.28	7/50	51/BCH	6/66.66	2/22.22	66-BCH
Imipenem	1/0.4	237/95.56	73-MCH	30/1.69	143/80.79	-	13/25.49	38/74.5	14-MCH	1/15.15	59/89.39	9-MCH
Levofloxacin	121/37.69	195/60.74	-	93/45.36	107/52.19	-	29/44.61	35/53.84	-	18/24.00	53/70.66	
Meropenem	1/0.40	241/97.17	73-MCH	30/1.69	144/81.35	28-MCH	13/25.49	38/74.5	14-MCH	0	62/93.93	9-MCH
Nitrofurantoin	46/14.33	194/60.43	-	48/23.41	53/25.85	-	4/28.57	7/50	51-BCH	3/33.33	4/44.44	66-BCH

n—number, %—percentage; R—resistant; S—sensitive; NA—not applicable; BCH—Burghele Clinical Hospital, Bucharest; MCH—Mures County Hospital.

**Table 4:** Overall antibiotic resistance profile in Gram-negative uropathogens.

Antibiotics	Gram-Positive Organism Isolated				
	*Enterococcus* spp.			*Staphylococcus* spp.	
	R·n/%	S·n/%	NA	R·n/%	S·n/%
Amikacin	-	-	-	2/4.0	46/92.0
Ampicillin	28/15.90	133/75.56	-	-	-
Sumetrolim-Sulfamethoxazole	-	-	-	18/36.0	26/52.0
Ceftazidime	-	-	-	9/18.0	35/70.0
Fosfomycin	5/2.84	154/87.5	-	-	-
Levofloxacin	74/42.04	92/52.27	-	27/54.0	21/42.0
Linezolid	2/1.28	141/90.38	20-MCH	-	-
Nitrofurantoin	7/3.97	151/85.79	-	-	37/74.0
Penicillin	59/33.52	105/59.65	-	33/66.0	8/16.0
Vancomycin	1/0.56	165/93.75	-	-	-

n—number, %—percentage; R—resistant; S—sensitive; NA—not applicable; MCH—Mures County Hospital.
